# Evaluation of a commercial coproantigen immunoassay for the detection of *Toxocara cati* and *Ancylostoma tubaeforme* in cats and *Uncinaria stenocephala* in dogs

**DOI:** 10.1007/s00436-022-07715-0

**Published:** 2022-11-17

**Authors:** Daniela Hauck, Katharina Raue, Katrin Blazejak, Rita M. Hanna, David A. Elsemore, Nikola Pantchev, Christina Strube

**Affiliations:** 1 Institute for Parasitology, Centre for Infection Medicine, University of Veterinary Medicine Hannover, Buenteweg 17, 30559 Hanover, Germany; 2 IDEXX Laboratories Inc, Westbrook, ME 04092 USA; 3 IDEXX Laboratories, Humboldtstrasse 2, 70806 Kornwestheim, Germany

**Keywords:** Roundworms, Hookworms, Endoparasites, Helminths, Canine, Feline, Antigen detection, ELISA, Diagnosis

## Abstract

Coproantigen immunoassays (IDEXX Fecal Dx® antigen tests) were evaluated for their ability to identify *Toxocara cati* and *Ancylostoma tubaeforme* infections in cats and *Uncinaria stenocephala* infection in dogs. Five cats were experimentally infected with 500 embryonated eggs of *T. cati*, eight cats with 500 third-stage larvae (L3) of *A. tubaeforme* and seven dogs with 500 L3 of *U. stenocephala*. In addition to the three coproantigen tests, the course of infection was monitored by a combined sedimentation-flotation method with ZnSO_4_ as flotation medium (specific gravity: 1.28–1.30) and a modified McMaster method in case of copromicroscopically positive samples. Eggs of *T. cati* were first observed between 28 and 54 days post infection (dpi) in four of the five infected cats. In these four cats, positive roundworm coproantigen signals were obtained between 16 and 44 dpi. Positive coproantigen signal always preceded egg observations, but the interval varied between 6 and 30 days. Hookworm-specific positive coproantigen signals were detected in seven of the eight *A. tubaeforme* infected cats between 10 and 52 dpi, while consecutive egg excretion was observed in three cats between day 26 and 54 pi. Of these three, coproantigen signal preceded egg observation by 12 to 24 days. Four cats had positive coproantigen results in the absence of egg excretion, and one cat never achieved a positive result for egg or coproantigen. In six of seven *U. stenocephala* infected dogs, infection was confirmed by copromicroscopy between 16 and 24 dpi as well as for hookworm coproantigen between 10 and 14 dpi. Coproantigen signal was detected prior to egg observation by 2 to 14 days. No cross-reactions between the roundworm, hookworm und whipworm tests occurred in study animals. The results of this study demonstrate the ability of the coproantigen tests to detect the common roundworm and hookworm infections in cats and *U. stenocephala* infections in dogs as well as the ability to detect the prepatent stage of infection.

## Introduction

Roundworms, hookworms and whipworms are among the most common intestinal helminths in cats and dogs worldwide (Abulude 2019; Drake et al. 2019; Fu et al. 2019; La Torre et al. 2018; Overgaauw and Nijsse 2020) and can cause serious health problems for pets and, in the case of the zoonotic roundworm and hookworm species, for their owners (Bowman et al. 2010; Ma et al. 2020; Strube et al. 2020a; Waindok et al. 2021). In cats, the roundworm *Toxocara cati* and the hookworm *Ancylostoma tubaeforme* are two to the most frequent parasitic nematodes, although reported prevalences (*T. cati*: 0.0–91.0%; *A. tubaeforme*: 0.5–91.0%) vary widely by region or epidemiological background of the cats (Anderson et al. 2003; Barutzki and Schaper 2011; Becker et al. 2012; Beugnet et al. 2014; Capari et al. 2013; Ketzis and Lucio-Forster 2020; Millán and Casanova 2009; Sommerfelt et al. 2006; Yamaguchi et al. 1996; Zibaei et al. 2007).

*Toxocara cati* has a complex biological cycle, based on its various transmission modes and larval migration routes in the feline definitive host, depending mainly on the source of infection and gestation status. Feline infections with *T. cati* occur by ingestion of embryonated eggs from the environment or paratenic hosts tissues harbouring infective third-stage larvae (L3) as well as lactogenic L3 transmission to the offspring if the queen acquires an acute infection during late pregnancy (Bowman 2009; Coati et al. 2004; Lee et al. 2010; Swerczek et al. 1971; Ziegler and Macpherson 2019). The prepatent period of *T. cati* is described to vary between 38 and 56 days (Bowman 2002). Clinical signs of toxocarosis occur usually in kitten and include diarrhoea, emesis, stunted growth and abdominal discomfort up to intestinal obstruction (Bowman 2009). In humans, zoonotic *T. cati* infections may result in visceral, ocular or neural *larva migrans* and associated signs of disease (Auer and Walochnik 2020; Strube et al. 2020b).

Feline hookworm infections with *A. tubaeforme* occur either by oral ingestion of L3 in the environment or infected paratenic hosts, or by percutaneous infection with L3 actively penetrating the skin. After 18–28 days post infection (dpi), worms enter patency (Okoshi and Murata 1967). Adult specimens of *A. tubaeforme* attach to the intestinal wall and feed on blood, which may result in anaemia, diarrhoea and weight loss in kittens. High burdens can even be fatal (Onwuliri et al. 1981).

Among the most common canine hookworm species are *Ancylostoma caninum* and *Uncinaria stenocephala*. In the colder climates of central and northern Europe, dogs are almost exclusively infected with *U. stenocephala* (Štrkolcová et al. 2022), whose life cycle is similar to that of *A. tubaeforme*, but oral ingestion of L3 from the environment is the predominant mode of infection. The prepatent period for *U. stenocephala* is usually 14–18 days, but it may require up to 4 weeks to reach patency (Nolan et al. 1992; Stoye 1983). Unlike *Ancylostoma* spp., *U. stenocephala* is a mucosal plug-feeder and ingests only small amounts of blood. Thus, it is less pathogenic, but heavy infections of puppies can cause diarrhoea, hypalbuminaemia and mild anaemia (Traversa 2012).

The control of intestinal nematode infections in dogs and cats to protect animal health and prevent zoonotic infections of humans is an important sector of veterinary care. To achieve effective control, common recommendations include either anthelmintic treatments or examination of faecal samples and treatment according to findings in regular intervals (CAPC 2020; ESCCAP 2020). In cats and dogs, common gastrointestinal nematode infections are frequently diagnosed by faecal egg detection using flotation methods. However, faecal flotation may not recover eggs at low parasite burdens or intermittent shedding and remains negative in single-sex or prepatent infections. Furthermore, coprophagy may lead to false-positive results. Methodological deficiencies such as small sample size, non-optimal flotation solution or an inexperienced examiner add to the biological factors compromising the sensitivity and specificity of faecal egg detection techniques.

To overcome these shortcomings and to complement copromicroscopic examinations, several other methods have been developed to aid in the detection of parasitic infections, including detection of parasite antigens in, e.g. serum or faeces. Such antigen tests have become an accepted routine diagnostic procedure for a number of parasitic infections in veterinary or human medicine, and in particular for intestinal protozoans such as *Giardia* or *Cryptosporidium* spp., a number of different tests are commercially available (Carlin et al. 2006; Johnston et al. 2003; Mekaru et al. 2007; Olson et al. 2010; Tanner et al. 1994; Weitzel et al. 2006).

Regarding intestinal nematodes in dogs, coproantigen immunoassays (IDEXX Fecal Dx® antigen tests) were developed for the simultaneous detection of hookworm (*Ancylostoma caninum*), roundworm (*Toxocara canis*) and whipworm (*Trichuris vulpis*) infections, recognizing specific excreted or secreted (E/S) antigens of (pre-)adult worms (Elsemore et al. 2017; Elsemore et al. 2014). In the case of hookworms, the test is based on polyclonal (pAb) and monoclonal antibodies (mAb) against recombinantly expressed *A. caninum* Asp-5 protein (Zhan et al. 2003). Capture antibodies are polyclonal, and detection antibodies are monoclonal immunoglobulin (Ig) G kappa subtypes. For detection of roundworms and whipworms, mAbs were developed against recombinantly expressed *T. canis* protease inhibitor homolog (Babin et al. 1984; Peanasky et al. 1984) and whipworm porin protein (Elsemore et al. 2017; Elsemore et al. 2014), respectively. Both capture and detection antibodies are monoclonal IgG kappa subtypes (Elsemore et al. 2017; Elsemore et al. 2014). Interestingly, a field study demonstrated that the *T. vulpis* test was able to detect the feline counterpart *Trichuris felis* (Geng et al. 2018). Furthermore, the *T. canis* test also appears to be suitable to detect *T. cati* coproantigen, as shown by initial investigations with field samples, i.e. naturally infected cats (Elsemore et al. 2017). This is not surprising since the *T. cati* protease inhibitor homologs show more than 95% identity with *T. canis* (Elsemore 2020; Elsemore et al. 2017).

However, the observed cross-reactivity of the IDEXX coproantigen tests with feline counterparts in field samples needs to be verified in experimentally infected cats under laboratory conditions. Moreover, the hookworm test is not yet validated for the detection of *U. stenocephala*, the most common hookworm in dogs in central and northern Europe. Therefore, this study aimed to evaluate the performance of the IDEXX Fecal Dx® antigen tests in experimental *T. cati* and *A. tubaeforme* infections in cats and experimental *U. stenocephala* infections in dogs.

## Materials and methods

### Experimental infections of cats and dogs

Samples analysed in this study were surplus samples from experimental infections of cats (*A. tubaeforme*, *T. cati*) and dogs (*U. stenocephala*) for parasite maintenance performed at the Institute for Parasitology, University of Veterinary Medicine Hannover, Germany. Experimental cat and dog infections have been permitted by the ethics commission of the Animal Care and Use Committee of the German Lower Saxony State Office for Consumer Protection and Food Safety (*Niedersächsisches Landesamt für Verbraucherschutz und Lebensmittelsicherheit*) under the reference number 33.19-42502-05-17A206.

Animal husbandry and handling complied with the European Commission guidelines for the accommodation of animals used for experimental and other scientific purposes. In brief, all animals were kept in pairs and received a standard commercial dry diet at recommended rates, water was provided *ad libitum*. Cats (European Shorthair) were housed indoors in rooms environmentally enriched with shelves, scratch poles and toys. Dogs (Beagles) had outdoor access on concrete floors during the daytime. Indoor and outdoor areas were equipped with raised platforms and toys; in addition, chewables were provided once or twice a week.

All animals used for experimental infections were owned by the Institute of Parasitology, University of Veterinary Medicine Hannover. Some of them had been previously infected for parasite maintenance, but not with the respective species used to generate the data described here.

Five cats were experimentally infected with *T. cati* (field isolate HannoverTcati2010) by oral administration of 500 embryonated eggs each. For experimental hookworm infections, eight cats were orally inoculated with 500 *A. tubaeforme* L3 (field isolate AlbaniaAt2011) each. Similarly, seven dogs were orally inoculated with 500 *U. stenocephala* L3 (field isolate HannoverUs2017) each. Additionally, one 6-month-old male dog was included in the study. This animal was not experimentally infected and served as a companion animal of an experimentally *U. stenocephala*-infected dog. Detailed information on the age and sex of the animals are provided in Tables 1, 2, and 3.

**Table 1:** Overview on egg excretion and *T. canis*-coproantigen immunoassay positivity in *T. cati* infected cats.

Animal ID	Sex	Age at infection	Sampling period (dpi)	Egg excretion (dpi)	Coproantigen positivity (dpi)
T.cati 1	Male	3 months	− 2 to 60	28–42, 48–60	16–60
T.cati 2	Male	3 months	− 2 to 70	54–70	24, 28–32, 36, 40–42, 52–70
T.cati 3*	Male	7 months	− 2 to 54	44–54	38, 44–54
T.cati 4*	Male	7 months	− 2 to 54	48–54	44–54
T.cati 5*	female	9 years	− 2 to 102	Not detected	Not detected

^*^Cats were pre-infected with *A. tubaeforme* (T.cati 3 = A.tub 5; T.cati 4 = A.tub 7; T.cati 5 = A.tub 1)

**Table 2:** Overview on egg excretion and *A. caninum*-coproantigen immunoassay positivity in *Ancylostoma tubaeforme* infected cats

Animal ID	Sex	Age at infection	Sampling period (dpi)	Egg excretion (dpi)	Coproantigen positivity (dpi)
A.tub 1	Female	9 years	− 2 to 56	26, 38–56	14–56
A.tub 2	Male	10 years	− 2 to 94	48–50, 54–64, 68–94	34–94
A.tub 3	Male	7 years	− 2 to 94	54, 56, 64–66, 72–84, 86–94	30–94
A.tub 4	Female	11 years	− 2 to 64	Not detected	52–60, 64
A.tub 5	Male	4 months	− 2 to 62	8*	30, 38–42
A.tub 6	Male	10 years	− 2 to 64	Not detected	20, 40, 46-52
A.tub 7	Male	4 months	− 2 to 62	Not detected	10, 16
A.tub 8	Female	12 years	− 2 to 56	Not detected	Not detected

^*^One egg detected with the combined sedimentation-flotation method, while the McMaster method remained negative

**Table 3:** Overview on egg excretion and coproantigen immunoassay positivity in *Uncinaria stenocephala* infected dogs

Animal ID	Sex	Age at infection	Sampling period (dpi)	Hookworm egg excretion (dpi)	*Ancylostoma caninum* coproantigen positivity (dpi)	*Toxascaris leonina* egg excretion (dpi)	*Toxocara canis* coproantigen positivity (dpi)
U.sten 1	Female	2 years	− 2 to 52	22–34, 38–48,52	14–52	Not detected	Not detected
U.sten 2	Male	5 months	− 2 to 37	18–37	10–37	Not detected	Not detected
U.sten 3	Female	4 years	− 1 to 30	26–30	10–30	Not detected	Not detected
U.sten 4	Male	5 years	− 1 to 30	20–30	10–30	Not detected	Not detected
U.sten 5*	Male	6 months	− 2 to 56	30*	20	Not detected	Not detected
U.sten 6	Female	2 years	− 2 to 53	Not detected	Not detected	Not detected	Not detected
U.sten 7**	Male	2 years	− 2 to 44	22-30, 38-44	14-44	38-44	36-44
U.sten 8**	Male	2 years	− 2 to 44	18-30, 38-44	18-44	38-44	38, 42

^*^Partner animal (not experimentally infected) of U.sten 2; hookworm eggs detected with the combined sedimentation-flotation method, while the McMaster method performed five times between 35 and 56 dpi remained negative

^**^Dogs were pre-infected with *Toxascaris leonina* in an unrelated experiment

### Faecal egg detection and egg counts

Individual faecal samples from each animal were collected every second day, starting 1 or 2 days before experimental infection. Faecal sampling was conducted at least until day 56 pi for *A. tubaeforme*, day 54 pi for *T. cati* and day 30 pi for *U. stenocephala*. All samples were examined at the Institute of Parasitology, University of Veterinary Medicine Hannover, with a combined sedimentation-flotation method using ZnSO_4_ as flotation solution (specific gravity [SG]: 1.28–1.30), as previously described by Becker et al. (2016). Egg positive samples were additionally subjected to a modified McMaster method using saturated NaCl flotation solution (SG: 1.20) (Becker et al. 2016) to determine the number of eggs per gram faeces (EpG). If it was not possible to examine the samples at the day of collection, e.g. at the weekend, samples were stored at 3-8 °C and processed within 2 days. Furthermore, 5 g of each sample was stored at − 20 °C and shipped to IDEXX Laboratories, Westbrook, USA, for coproantigen testing.

### Coproantigen immunoassay testing

The faecal samples were analysed at IDEXX Laboratories Inc., Westbrook, USA, using the commercially available coproantigen immunoassay for detection of roundworm, hookworm and whipworm infection in dogs (IDEXX Fecal Dx® antigen tests). Coproantigen testing was performed as previously described (Elsemore et al. 2017; Elsemore et al. 2014). Finally, plates were read at a wavelength of 650 nm within 10 min after addition of stop solution. For data analysis, a cut-off of 0.100 optical density (OD) was used (Elsemore et al. 2017; Elsemore et al. 2014).

To test for cross-reactivity among the three nematode families, all faecal samples were tested with all three, i.e. roundworm, hookworm and whipworm coproantigen tests.

## Results

### Detection of *Toxocara cati*

**Fig. 1:**
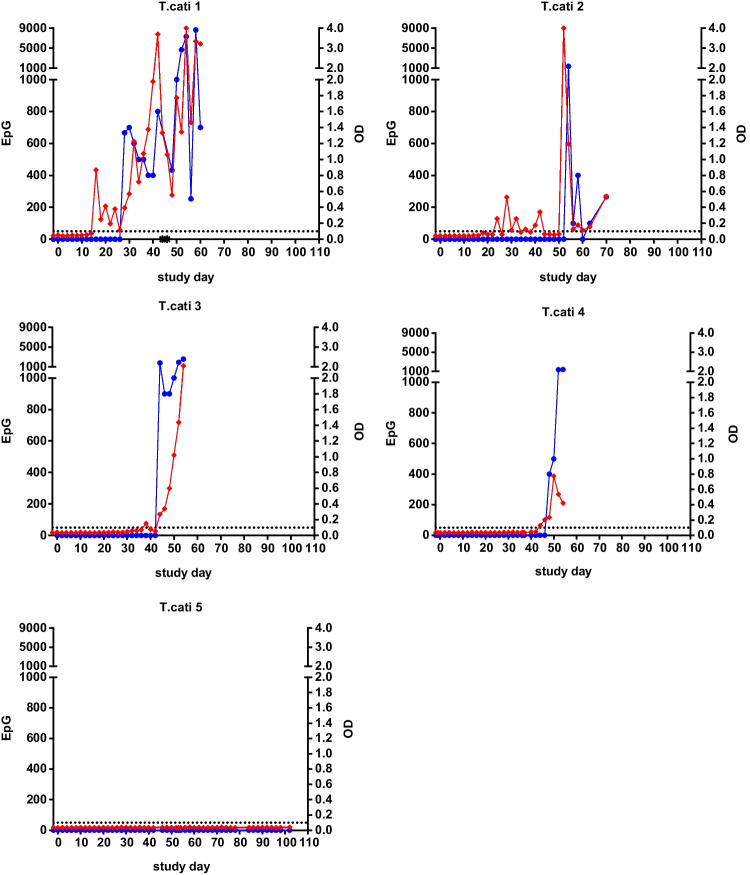
Results of faecal egg counts (EpGs) and coproantigen detection (OD) in five cats experimentally infected with *Toxocara cati*. Note that the sampling period differed between individual cats. Blue dots: *T. cati* EpG; red squares: *T. cati* OD; black asterisks: egg detection by combined sedimentation-flotation. The dotted black horizontal line marks the cut-off of 0.100 OD

Excretion of *T. cati* eggs was copromicroscopically confirmed in four of the five experimentally infected cats, starting between day 28 and 54 pi (Table 1). Coproantigen detection revealed positive signals between day 16 and 44 pi, which was 6 to 30 days earlier than the first egg detection in the respective animal. One cat (*T. cati* 5) remained negative for both eggs and coproantigen throughout the observation period. Figure 1 displays individual egg counts and *T. cati* coproantigen results over the individual sampling period.

Hookworm and whipworm coproantigen testing of these samples was negative demonstrating no cross-reactivity between nematode tests.

### Detection of *Ancylostoma tubaeforme*

**Fig. 2:**
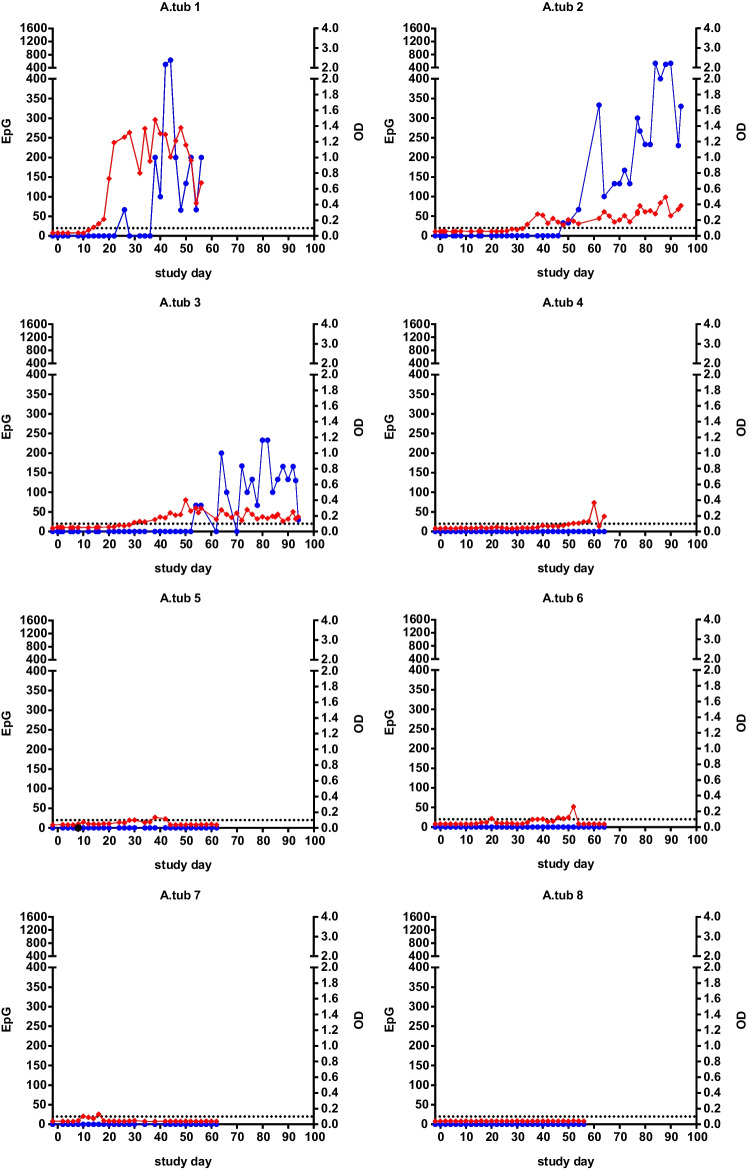
Results of faecal egg counts (EpGs) and coproantigen detection (OD) in eight cats experimentally infected with *Ancylostoma tubaeforme*. Note that the sampling period differed between individual cats. Blue dots: *A. tubaeforme* EpG; red squares: *A. tubaeforme* OD. The dotted black horizontal line marks the cut-off of 0.100 OD

*A. tubaeforme* egg excretion was observed in four of the eight experimentally infected cats. In three animals, egg shedding was observed over a period of time starting between day 26 and 54 pi (Table 2); however, two of these animals showed intermittent EpG values of 0. In one cat, a single *A. tubaeforme* egg was detected once at day 8 pi in the combined sedimentation-flotation, while the McMaster method and further faecal examinations remained negative. In the other four cats, no egg excretion was observed. In contrast, seven cats were defined as positive by the *A. caninum* coproantigen test. In six cats, coproantigen was detected between day 10 and 52 pi, and thus 12 to 24 days before the first egg observation. An exception was cat A.tub 5, where a single egg was detected once on day 8 pi and the first positive coproantigen signal at day 38 pi. In one animal (A.tub 8), neither copromicoscopy nor the coproantigen test yielded positive results. The course of EpG and *A. tubaeforme* coproantigen signal (OD) values for the individual cats is shown in Fig. 2.

Again, no cross-reactivity was noted on the roundworm or whipworm coproantigen tests for the duration of the study.

### Detection of *Uncinaria stenocephala*

In six of the seven experimentally infected dogs, excretion of *U. stenocephala* eggs started between day 16 and 24 pi (Table 3). In these dogs, the *A. caninum* coproantigen test resulted in positive signals between day 10 and 14 pi, i.e. 2 to 14 days before the first copromicroscopic egg detection. Again, one animal remained negative with both techniques throughout the observation period.

Another dog (U.sten 5) was not experimentally inoculated with *U. stenocephala*, but was housed with dog U.sten 2 as a partner animal. Nevertheless, hookworm eggs were detected by sedimentation-flotation on day 30 pi in its faecal sample, and 10 days before (day 20 pi), it exceeded the cut-off value in the *A. caninum* coproantigen test once.

Furthermore, despite previous deworming and negative copromicroscopic examinations before experimental *U. stenocephala* infection, a reemergence of an earlier experimental *T. leonina* infection (inoculation dose: 200 embryonated eggs) was detected in two dogs (U.sten 7 and U.sten 8) by both copromicroscopic examination and the *T. canis*-specific immunoassay reagents. Positive coproantigen detection and egg excretion started simultaneously in one dog, whereas in the other dog, the coproantigen test turned positive 2 days before the first egg detection.

**Fig. 3:**
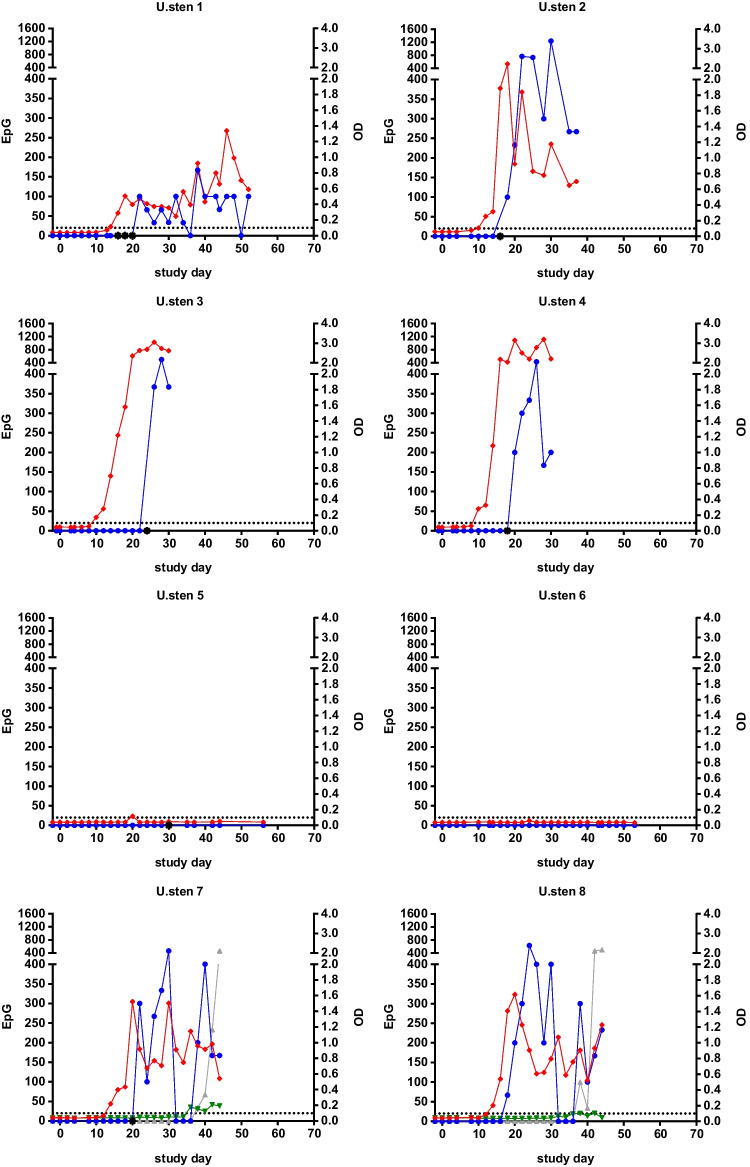
Results of faecal egg counts (EpG) and coproantigen detection (OD) in eight dogs experimentally infected with *Uncinaria stenocephala*. Additionally, values for *Toxascaris leonina* are shown for the two dogs coinfected with this parasite. Note that the sampling period differed between individual dogs. Blue dots: *U. stenocephala* EpG; red squares: *U. stenocephala* OD; grey triangles: *T. leonina* EpG; green triangles: *T. leonina* OD; black asterisks: egg detection by combined sedimentation-flotation. The dotted black horizontal line marks the cut-off of 0.100 OD

Individual egg counts and coproantigen values (OD) over the observation period are shown in Fig. 3. The whipworm test was negative for all dogs for the duration of the study. The roundworm test was negative for all dogs except the two animals that demonstrated re-emergence of previous *T. leonina* infection.

## Discussion

A coproantigen test to diagnose ascarid, hookworm and whipworm infections in dogs and cats is commercially available. One main feature of testing for an excreted/secreted antigen in faeces is the potential to identify the presence of nematodes even in the absence of detectable egg excretion, e.g. in the prepatency period or intermittent shedding, while preventing false positive results due to spurious eggs through, e.g. coprophagy, grooming or contaminated drinking water. To date, the coproantigen immunoassay is tested only for the above-mentioned parasites in experimentally infected dogs, but its applicability to related parasites, also in experimentally infected cats, would be desirable. Studies with field infections showed the tests were useful for detection of *T. cati* (Elsemore et al. 2017) and *T. felis* (Geng et al. 2018) in feline samples, attributed to closely related antigen homologs. Here, we evaluated the capability of coproantigen tests, to detect *T. cati* and *A. tubaeforme* infections in cats and *U. stenocephala* infections in dogs over a time course.

In all experimentally infected cats with a patent infection, the hook- and roundworm coproantigen tests turned positive. Furthermore, coproantigen detection was able to detect infections in the prepatent period, resulting in positive signals 4–30 days before detectable egg shedding for *T. cati* and 12–24 days for *A. tubaeforme* infected cats, indicating the test’s capability to detect E/S antigen of immature or young adult worms in the intestine. Such prepatent detection was also previously reported for coproantigen detection of *A. caninum*, *T. canis* and *T. vulpis* in dogs (Elsemore et al. 2017; Elsemore et al. 2014). The only exception from prepatent detection was observed for cat A.tub 5, where one hookworm egg was detected once with the sedimentation-flotation method 8 dpi, while the positive hookworm ELISA signals were observed at low levels on 30 and 38–42 dpi only. However, the prepatent period of *A. tubaeforme* is typically between 18 and 28 dpi; thus, an accidental contamination has to be considered (Okoshi and Murata 1967). This is supported by the fact that no eggs were detected in the subsequent McMaster method on 8 dpi nor by the sedimentation-flotation method until 62 dpi. Another notable finding was that the hookworm test detected coproantigen in four infected cats (cat A.tub 4, 6 and 7), which were copromicroscopically negative through the whole observation period. This may be related to the age of the cats (ten and eleven years of A. tub 6 and 4, respectively), resulting in low parasite burdens with no or very low egg excretion below the detection limit of the sedimentation-flotation method, or single-sex infections, which cannot be diagnosed by faecal flotation. However, a 4-month-old cat (A.tub 7) was constantly negative during faecal examination. A common feature in these three cats was low hookworm test OD values, which might reflect such low or single-sex infections, although it cannot be entirely ruled out that the hookworm ELISAs were false positive, or the applied cut-off was too low. Negative copromicroscopically and coproantigen results were observed in one *A. tubaeforme* (A.tub 8) and one *T. cati* (T.cati 5) infected cat. The mode of failure is unknown, but it is possible that inoculation of larvae led to a somatic migration where egg laying and coproantigen tests would be expected to be negative. Of note, all animals that remained coproscopically negative throughout the study (including dog U. sten6, see below) had not been previously infected for endoparasite maintenance, thus ruling out potential cross-immunity as the reason for the lack of detection.

The hookworm coproantigen test also detected all dogs with patent experimental infection of *U. stenocephala*. Coproantigen detection occurred between 2 and 14 days before egg detection. Positive results were not observed for eggs or coproantigen in one dog (U. sten6), presumably due to an abortive infection or somatic larvae migration. Interestingly, one dog, U.sten 5, which was group-housed with U.sten 2 but not experimentally infected, exhibited a positive coproantigen result at 20 dpi, and eggs were detected by the sedimentation-flotation method at 30 dpi. Coprophagy could be a possible explanation. Elsemore (2020) reported that coprophagy can result in a false positive antigen signal in an experimental model, even if the window of false antigen detection is short. This situation would most likely lead to false-positive results for both egg and coproantigen testing.

In two 2-year-old dogs (U.sten 7 and 8), previous infections with the roundworm *T. leonina* re-emerged in addition to the experimental hookworm infection of the present study, despite deworming and negative copromicroscopic results before the start of the study. Granted that the anthelmintic used once (100 mg/kg BW fenbendazole (Panacur® 500 mg, MSD Animal Health) as recommended by the manufacturer for single treatment in adult dogs) was effective, larvae sequestered in tissue at the time of treatment could have repopulated the intestinal lumen of these dogs, leading to renewed egg excretion about 7 weeks after deworming. Roundworm coproantigen was detected 2 days before onset of *T. leonina* egg excretion in one dog and remained above the cut-off, albeit at low level, until the end of sample collection 8 days later. In the other dog, a positive roundworm coproantigen signal was observed twice: concurrently with the first day of *T. leonina* egg detection and again 4 days later. Positive signals were at a low level even though > 400 EpG was reached at the end of the study. This observation might indicate a low *T. leonina* burden after deworming, potentially due to intestinal repopulation by somatic larvae.

Overall, it was confirmed that the hookworm and roundworm coproantigen tests were able to detect the feline counterparts (i.e. *A. tubaeforme* and *T. cati*), as previous investigations of field samples indicated (Elsemore et al. 2017). Furthermore, it could be demonstrated that the hookworm test reliably detects coproantigen not only from *A. caninum* but also from *U. stenocephala*, the dominant hookworm species of dogs in central Europe. Additionally, it can be assumed that the roundworm test developed can also be used to recognize infections with the *T. leonina*. Noteworthy, no cross-reactivity between the three most common nematode groups in cats or dogs occurred for any of the samples. Even though the coproantigen tests cannot completely replace routine copromicroscopic examinations because the menu does not include other intestinal parasites (e.g. coccidia, tapeworms or rare nematodes. e.g. *Strongyloides* or *Capillaria* spp.), it is a useful tool to detect the most common intestinal dog parasites and has the potential to be expanded to cats as shown in the present study. Furthermore, coproantigen detection is helpful to identify false positive (coprophagia) or false negative (dehydration of the faecal sample or sampling errors, e.g. too small sample size or attached cat litter) copromicroscopic results (Elsemore 2020; Elsemore et al. 2017; Elsemore et al. 2014; Little et al. 2019). An advantage is the tests’ capability to detect infections prior to onset of patency, as observed in the present study for almost all study animals. Consequently, early anthelmintic treatment could prevent maturation of adult stages and egg excretion, thus reducing the infection risk of animals and humans due to environmental contamination.

## Data Availability

Data supporting reported results is contained within the article.
